# Molecular hydrogen-rhodiola as an adjuvant therapy for ischemic stroke in internal carotid artery occlusion: A case report

**DOI:** 10.1515/med-2025-1290

**Published:** 2025-10-15

**Authors:** Feng-Hao Chang, Jeng-Wei Lu, Chun-Chih Hu, Wun-Long Jheng, Shuk-Man Ka, Shinn-Zong Lin, Dueng‐Yuan Hueng, Yueh-Feng Sung, Ying-Hsuan Tsai, Jou-I Tu, Yi-Jung Ho, Kuang‐Yih Wang, Feng-Cheng Liu

**Affiliations:** Department of Internal Medicine, Tri-Service General Hospital, National Defense Medical University, Taipei, Taiwan; Department of Bioscience and Biotechnology, National Taiwan Ocean University, Keelung, Taiwan; Biotech Research and Innovation Centre, University of Copenhagen, Copenhagen, Denmark; The Finsen Laboratory, Rigshospitalet/National University Hospital, Faculty of Health and Medical Sciences, University of Copenhagen, Copenhagen, Denmark; Cancer Center, Hualien Tzu-Chi Hospital, Buddhist Tzu-Chi Medical Foundation, Hualien, Taiwan; Graduate Institute of Aerospace and Undersea Medicine, Department of Medicine, National Defense Medical University, Taipei, Taiwan; Department of Neurological Surgery, Tzu Chi Hospital, Hualien, Taiwan; Department of Neurological Surgery, Tri‐Service General Hospital, National Defense Medical University, Taipei, Taiwan; Department of Neurology, Tri-Service General Hospital, National Defense Medical University, Taipei, Taiwan; School of Pharmacy, National Defense Medical University, Taipei, Taiwan; Graduate Institute of Life Sciences, National Defense Medical University, Taipei, Taiwan; Rheumatology/Immunology and Allergy, Department of Internal Medicine, Tri-Service General Hospital, National Defense Medical University, Taipei, Taiwan

**Keywords:** case report, ischemic stroke, internal carotid artery occlusion, molecular hydrogen, rhodiola rosea, immune modulation

## Abstract

**Objectives:**

Acute ischemic stroke caused by internal carotid artery (ICA) occlusion carries high risks of disability and death. While treatments such as thrombolysis, mechanical thrombectomy, and revascularization offer benefits, many patients experience limited recovery. Molecular hydrogen, with its antioxidant and anti-inflammatory properties, shows promise as a neuroprotective agent. This case report explores the adjunctive use of molecular hydrogen–rhodiola therapy in a patient with ICA occlusion, with a focus on immune modulation and clinical outcomes.

**Case presentation:**

A 68-year-old male with a history of paroxysmal atrial fibrillation presented with left-sided hemiplegia and was diagnosed with right ICA occlusion (NIH Stroke Scale: 12; modified Rankin Scale: 5). Endovascular thrombectomy was attempted but unsuccessful (modified Thrombolysis in Cerebral Infarction score = 0). The patient subsequently underwent superficial temporal artery to middle cerebral artery bypass surgery. Postoperatively, he was initiated on daily molecular hydrogen-rhodiola capsule therapy. Serial immunological assessments demonstrated a progressive increase in type 1 regulatory T (Tr1) cells and regulatory B cells, along with enhanced T-cell immunoglobulin and mucin-domain containing-3 (TIM-3) expression on cytotoxic T (Tc) cells. Clinically, the patient exhibited marked neurological recovery, with motor strength improving from Medical Research Council grade 1 to grade 5 in the affected limbs over six months. Notably, steroid therapy was discontinued without relapse, and no adverse events were observed.

**Conclusion:**

This case highlights the potential of molecular hydrogen-rhodiola therapy as a safe and effective adjunctive treatment for ischemic stroke due to ICA occlusion. Notable improvements in immune modulation and motor function support its possible role in neurovascular recovery.

## 1 Introduction

Acute ischemic stroke resulting from internal carotid artery (ICA) occlusion poses a formidable clinical challenge, with reported mortality rates ranging from 25 to 30% within the first 24 h to nearly 60% by 1 month post-onset [1]. Despite advancements in acute stroke interventions including intravenous thrombolysis, mechanical thrombectomy, and bypass procedures, neurological recovery is often incomplete, and many patients are left with long-term disability [2,3]. The pathophysiology of ischemic stroke is multifactorial, involving oxidative stress, neuroinflammation, and immune dysregulation, which contribute not only to the initial ischemic insult but also influence subsequent reparative processes [4].

In recent years, extracranial-to-intracranial (EC–IC) bypass surgery has reemerged as a treatment option for carefully selected patients with ICA occlusion who are either ineligible for, or have failed, conventional endovascular interventions [5]. Nevertheless, even successful revascularization does not guarantee full neurological restoration, underscoring the urgent need for effective adjuvant therapies that can support neuroregeneration and improve functional outcomes [6].

Molecular hydrogen (H_2_) has garnered increasing attention as a therapeutic agent due to its selective antioxidant properties. H_2_ can neutralize cytotoxic hydroxyl radicals without disrupting physiological signaling by essential reactive oxygen species [7,8]. Preclinical and clinical studies have demonstrated that H₂ exerts anti-inflammatory, anti-apoptotic, and immunomodulatory effects across a range of pathological conditions [9,10,11]. In models of cerebral ischemia, H_2_ has been shown to confer neuroprotection via several mechanisms, including attenuation of oxidative damage, preservation of mitochondrial integrity, and regulation of inflammatory signaling pathways [12,13]. In addition, *Rhodiola rosea* L., a widely recognized botanical adaptogen, has been shown to exert protective effects against inflammation-related damage in various diseases, including cardiovascular disorders, neurodegenerative conditions, diabetes, sepsis, and cancer [14]. Its beneficial properties have been attributed to its antioxidative [15], immunomodulatory [16], and neuroprotective activities, along with its ability to modulate neurodegenerative processes [17,18]. Salidroside, an active compound isolated from the Tibetan medicinal herb Rhodiola rosea, has shown therapeutic potential in tuberculosis treatment. *In vivo* evidence indicates that it enhances host anti-mycobacterial immunity by stimulating the production of inflammatory cytokines [19].

The immune system plays a central role in both the acute and recovery phases of ischemic stroke. While early pro-inflammatory responses exacerbate neuronal injury, regulatory immune cells such as regulatory T cells (Tregs) and regulatory B cells (Bregs) are instrumental in resolving inflammation and facilitating tissue repair [20]. Emerging evidence suggests that H_2_ therapy may modulate these immune cell subsets, potentially promoting a more balanced immune milieu conducive to neural recovery [21,22].

In this case report, we describe a 68-year-old male with right ICA occlusion who received molecular hydrogen-rhodiola therapy as an adjuvant intervention following failed endovascular thrombectomy and subsequent EC–IC bypass surgery. We provide comprehensive immunological data demonstrating dynamic changes in key immune cell populations during hydrogen therapy, and we correlate these findings with the patient’s notable clinical improvement.


**Informed consent:** Written informed consent was obtained from the patient for publication of this case report.
**Ethical approval:** This study was approved by the Institutional Review Board (IRB) of Tri-Service General Hospital, National Defense Medical Center, Taiwan (IRB No. C202405219, approval date: July 31, 2024). All procedures were conducted in accordance with institutional guidelines and the ethical standards of the 1964 Declaration of Helsinki and its subsequent amendments.

## 2 Case presentation

A 68-year-old male with a medical history of paroxysmal atrial fibrillation, managed with apixaban (ELIQUIS) 5 mg twice daily, presented to the emergency department on November 12, 2024, at 07:00, with sudden-onset left-sided weakness. His past medical history was notable for hypertension, hyperlipidemia, and chronic hepatitis B virus carrier status. The patient had previously been independent in activities of daily living. Upon evaluation, neurological examination revealed leftward gaze limitation, central-type left facial palsy, left hemiplegia (Medical Research Council grade 1), and left-sided neglect.

**Figure 1:**
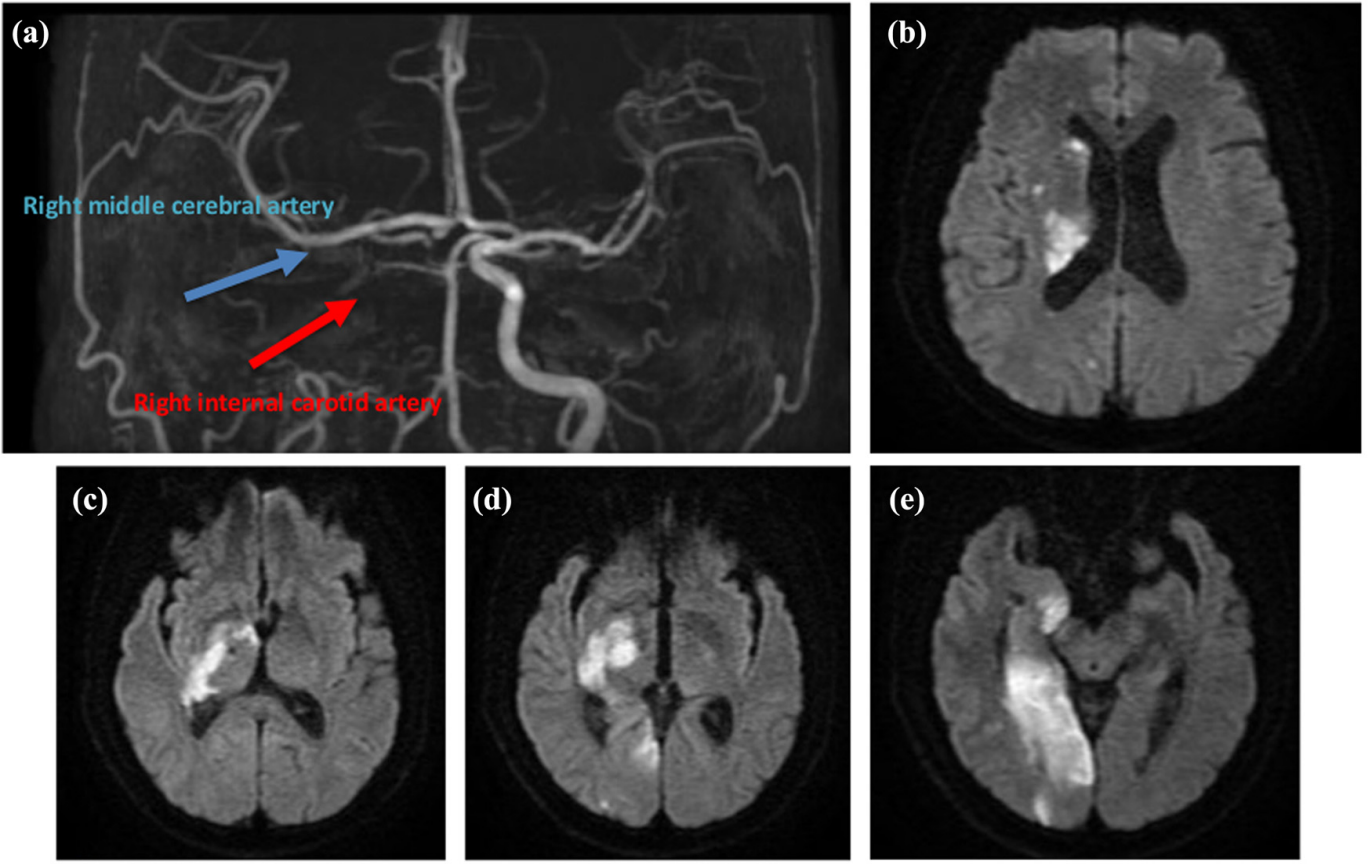
Initial neuroimaging findings. (a) Time-of-flight (TOF) MRA demonstrates occlusion of the right ICA and the right MCA, corresponding to the infarct distribution observed on MRI. (b–e) Diffusion-weighted brain MRI reveals acute infarcts involving the posterior limb of the right internal capsule, caudate head, hippocampus, and occipital lobe. Additional embolic infarcts are noted in the right cerebral hemisphere, along with two cerebral microbleeds. Blue arrow: The right MCA. Red arrow: The left ICA.

**Figure 2:**
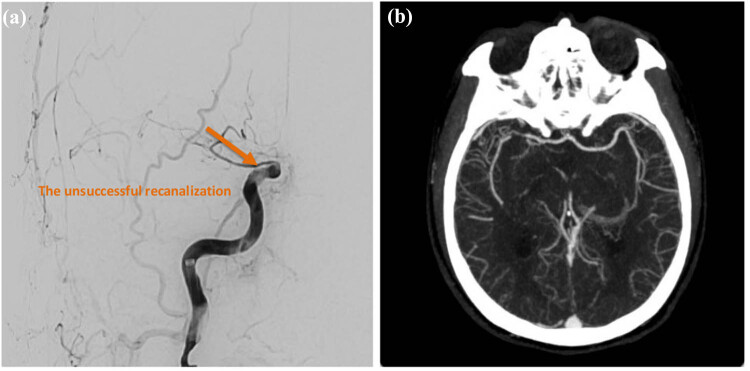
Endovascular thrombectomy findings. (a) Digital subtraction angiography reveals acute occlusion of the distal segment of the right ICA. (b) Post-procedural angiography demonstrates unsuccessful recanalization, with a mTICI score of 0, indicating persistent occlusion. Infarction involving the basal ganglia is also noted. Orange arrow: The orange arrow denotes an area of unsuccessful recanalization.

Initial non-contrast brain computed tomography and computed tomography angiography revealed hypodensities in the posterior limb of the right internal capsule, along with occlusion of the right distal ICA involving the petrous and cavernous segments (Figures 1 and 2). The middle cerebral artery (MCA) M1 and M2 segments were patent, although with reduced distal flow. The patient’s initial National Institutes of Health Stroke Scale (NIHSS) score was 12, and the modified Rankin Scale score was 5.

Intravenous thrombolysis with recombinant tissue plasminogen activator (rt-PA) was contraindicated due to ongoing anticoagulant therapy with apixaban (ELIQUIS) and a delayed presentation beyond the 4.5-h therapeutic window. The patient subsequently underwent emergency endovascular thrombectomy. The procedure involved two aspiration attempts, followed by three additional attempts using a stent retriever. Despite these interventions, successful recanalization was not achieved, with a final modified Thrombolysis in Cerebral Infarction (mTICI) score of 0. On November 13, 2024, brain magnetic resonance imaging (MRI) demonstrated acute infarcts involving the posterior limb of the right internal capsule, caudate head, hippocampus, and occipital lobe, along with additional embolic infarctions in the right frontal and parietal lobes. Magnetic resonance angiography (MRA) confirmed occlusion of the right ICA at the cavernous segment, as well as occlusion of the right fetal-type posterior cerebral artery (Figure 2).

**Figure 3:**
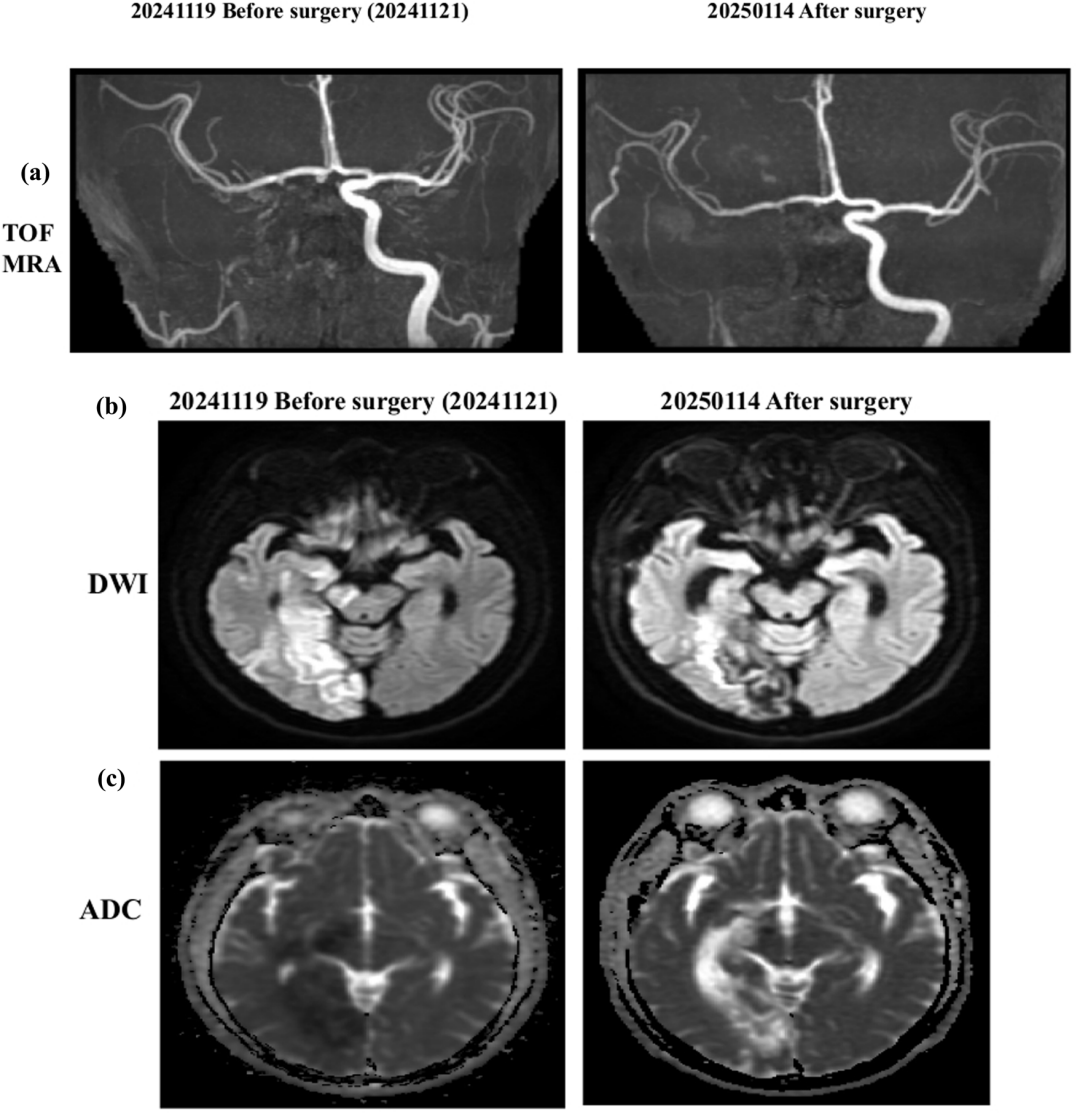
Serial neuroimaging before and after STA–MCA bypass surgery. (a) TOF MRA performed on November 19, 2024 (preoperative), demonstrates occlusion of the right ICA with reduced distal perfusion. Follow-up MRA on January 14, 2025 (postoperative), shows improved collateral circulation in the MCA territory, consistent with successful revascularization via STA–MCA bypass. (b) Diffusion-weighted imaging (DWI) prior to surgery reveals hyperintense signals in the right hemisphere, indicating acute ischemic changes. Postoperative DWI shows no new hyperintensities, suggesting the absence of additional infarcts. (c) Apparent diffusion coefficient (ADC) maps demonstrate corresponding preoperative hypointensities, confirming restricted diffusion in infarcted regions. Postoperative ADC findings reflect infarct maturation without evidence of extension.

**Figure 4:**
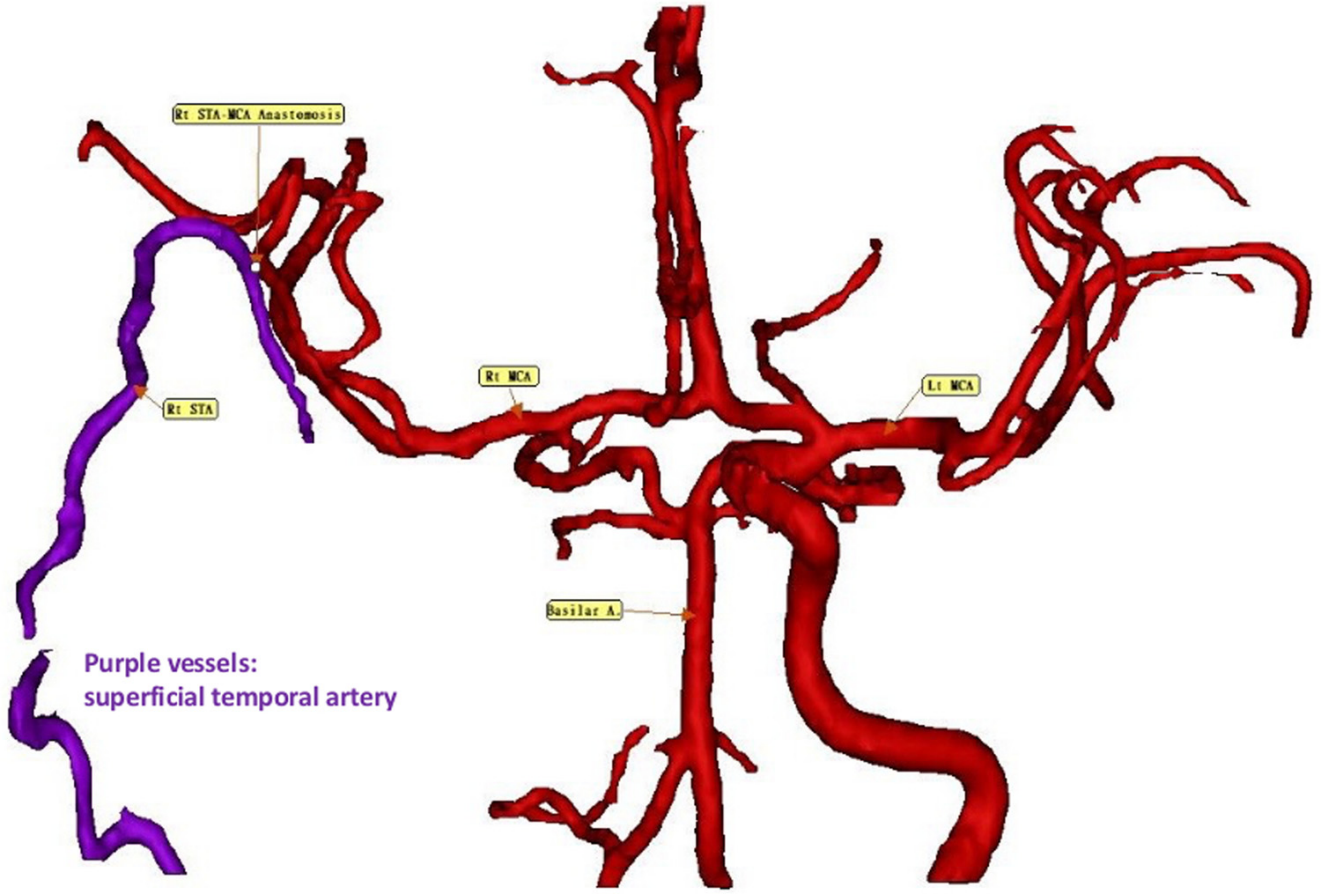
Three-dimensional reconstructions of the cerebral vasculature. Follow-up imaging on January 14, 2025, after STA–MCA bypass surgery, reveals improved cerebral perfusion. No new ischemic lesions are observed, confirming the absence of perioperative infarcts. Purple vessels: STA.

On November 21, 2024, the patient underwent a superficial temporal artery to middle cerebral artery (STA–MCA) bypass procedure (Figures 3 and 4). The initial postoperative course was uneventful; however, substantial neurological deficits persisted despite surgical revascularization. As part of adjunctive therapy, molecular hydrogen supplementation was initiated from November 13, 2024, to January 13, 2025 (2 months). This was followed by 10 days of one hydrogen with rhodiola capsule daily (PURE HYDROGEN, HoHo Biotech Co., Ltd., Taipei, Taiwan), each containing 170 mg of hydrogen-rich coral calcium, corresponding to approximately 1.7 × 10^21^ molecules of molecular hydrogen), after which a third blood test was conducted. The hydrogen with rhodiola therapy was then extended for an additional 2 months (PURE HYDROGEN), each containing 170 mg of hydrogen-rich coral calcium, corresponding to approximately 1.7 × 10^20^ molecules of molecular hydrogen, and 100 mg of rhodiola rosea extract.

Cerebral vascular imaging before surgery confirmed right ICA occlusion prior to the procedure. On November 13, 2024, postoperative imaging following STA–MCA bypass demonstrated enhanced cerebral perfusion, with no evidence of new ischemic lesions, indicating the absence of perioperative infarction (Figure 4). On January 13, 2025, adjunctive therapy with hydrogen–rhodiola capsules was administered. On March 13, 2025, after two months of adjuvant therapy with hydrogen-rhodiola capsules, postoperative imaging demonstrated improved cerebral perfusion, correlating with the notable neurological recovery of the patient. Specifically, motor function improved from severe left-sided weakness (Medical Research Council grade 1) to near-complete recovery, with MRC grade 5 in the left upper limb and grade 4 in the left lower limb.

**Figure 5:**
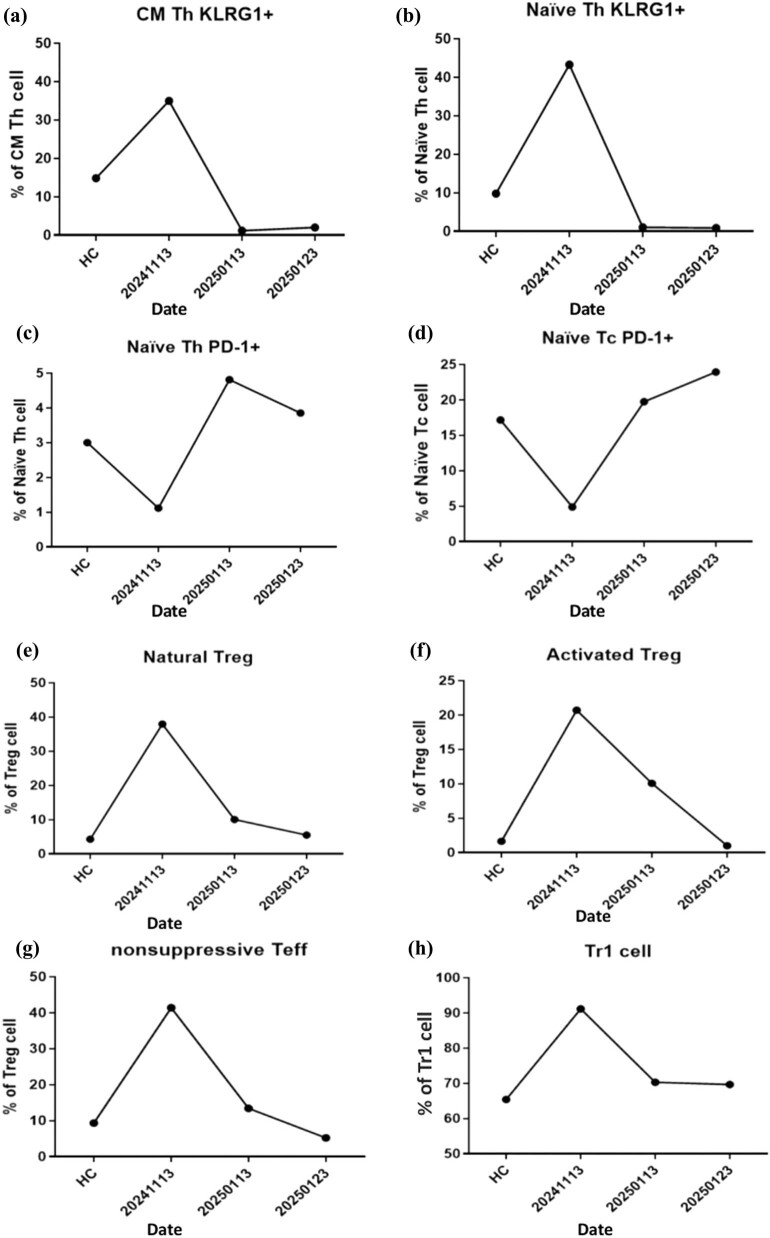
Dynamic changes in regulatory and exhausted T-cell subsets during hydrogen therapy alone or in combination with rhodiola. (a) Central memory CD4⁺ T helper (CM Th) cells expressing KLRG1 were elevated at baseline and decreased progressively during recovery. (b) Naïve Th cells expressing KLRG1 demonstrated a marked increase during the acute phase of stroke, followed by a significant decline post-treatment. (c) PD-1⁺ naïve Th cells decreased during the acute event but gradually increased after hydrogen therapy, reflecting restoration of immune checkpoint activity. (d) A similar trend was observed in PD-1⁺ naïve cytotoxic T cells (Tc). (e) Natural regulatory T cells (Tregs; CD127^low^FOXP3^⁺^) were initially elevated and normalized over time. (f) Activated Treg cells (CD45RA⁻FOXP3^high^) increased following hydrogen therapy. (g) Non-suppressive CD4⁺ effector T cells (CD25⁺FOXP3^low^) declined with treatment. (h) Type 1 regulatory T cells (Tr1; IL-10⁺FOXP3⁻) exhibited a sustained increase after therapy initiation. Health control (HC) group.

**Figure 6:**
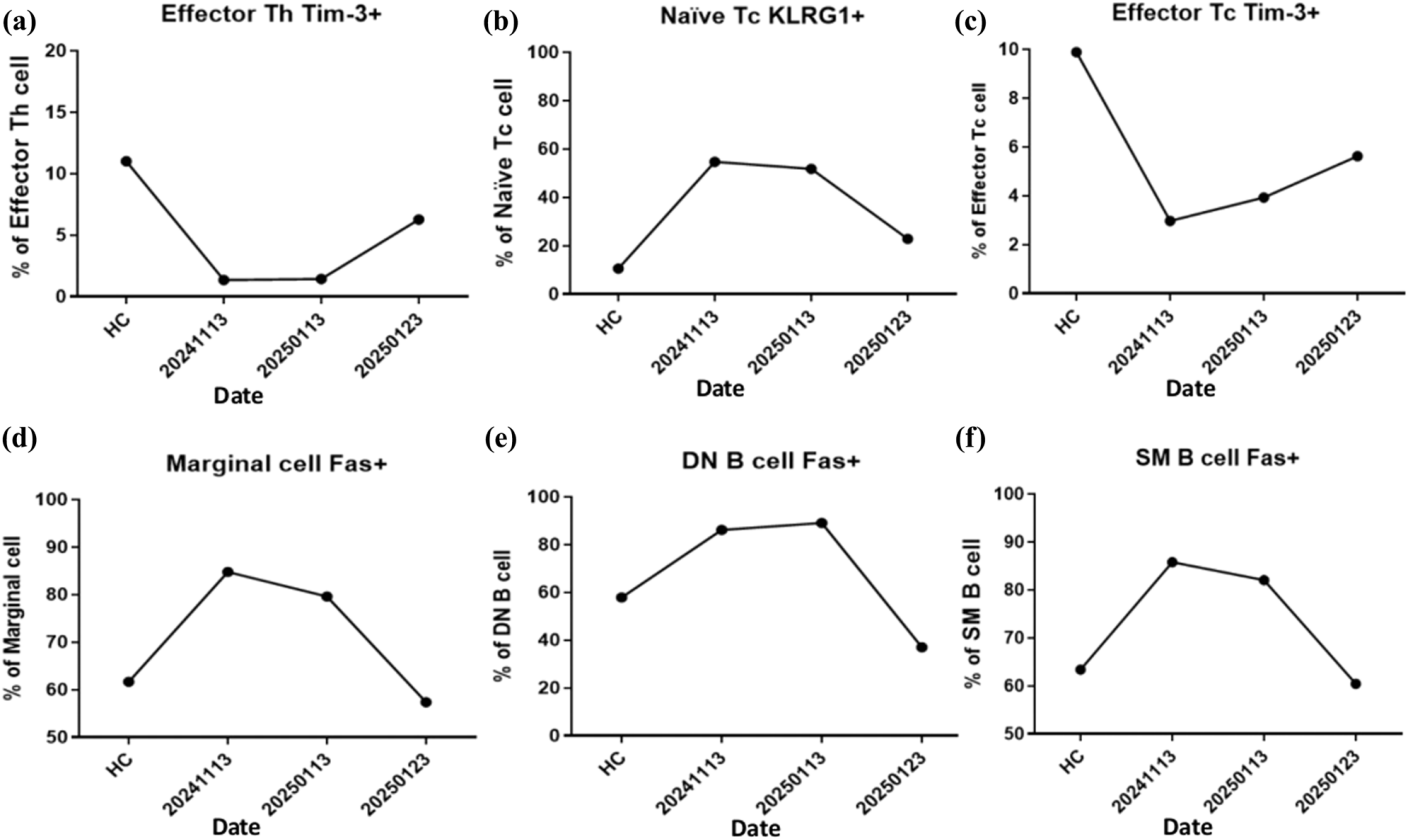
Immune checkpoint and apoptotic regulation following hydrogen therapy alone or in combination with rhodiola. (a) TIM-3 expression on effector CD4⁺ T helper (Th) cells increased progressively over the treatment period. (b) Naïve CD8⁺ cytotoxic T cells (Tc) expressing KLRG1 were elevated at baseline but declined steadily after treatment. (c) TIM-3 expression on effector Tc cells increased following hydrogen therapy. (d) Fas expression on marginal zone B cells was markedly elevated during the acute ischemic phase and decreased after therapy. (e) A similar pattern was observed in Fas⁺ DN B cells (IgD⁻CD27⁻). (f) Fas⁺ SM B cells also exhibited decreased expression levels post-therapy. Health control (HC) group.

**Figure 7:**
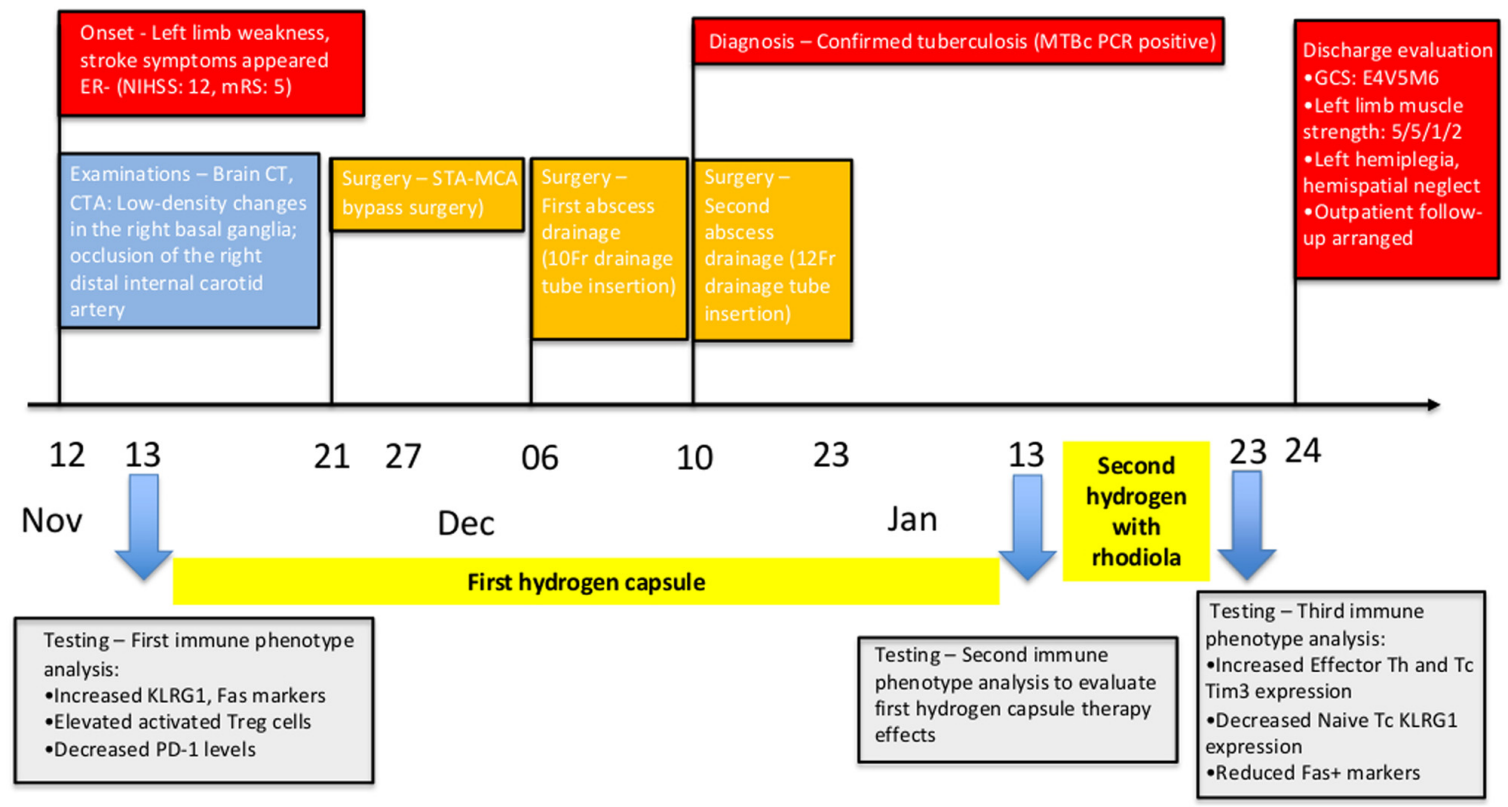
Clinical timeline and immunological evolution. This figure presents a chronological overview of key clinical events alongside dynamic immunophenotyping data across three major phases: (1) Initiation of hydrogen capsule supplementation during an arterial embolism episode, (2) postoperative recovery following STA–MCA bypass surgery, and (3) combined supplementation with hydrogen and Rhodiola capsules. Immunological trends observed over time include a progressive increase in regulatory Tr1-cell frequency and TIM-3 expression, along with a marked decrease in Fas⁺ apoptotic B-cell subsets. These immune changes temporally correlate with the patient’s gradual neurological recovery and overall clinical improvement.

In addition to the clinical and imaging data, immunophenotypic analysis and cell gating strategy were conducted in accordance with previously established protocols [22,23,24,25,26,27]. Flow cytometry was employed to evaluate immunological alterations in whole blood samples obtained before and after the administration of molecular hydrogen or hydrogen–rhodiola therapy. Sample preparation for subsequent analyses followed standard protocols, utilizing fluorescent dye-based labeling techniques in conjunction with commercially available dried antibody reagent kits (Beckman Coulter, USA). The procedures for sample processing, immunophenotypic profiling, and cell population gating were conducted in accordance with established methods previously described in the literature [28]. See supplementary methods for details. Immunophenotyping was performed three times (November 13, January 13, and January 23) to evaluate the patient’s immunological response to the adjunctive hydrogen-rhodiola therapy. The analyses demonstrated progressive immunomodulatory changes, including increased expression of the regulatory marker T-cell immunoglobulin and mucin-domain containing-3 (TIM-3) and a reduction in Fas-positive (Fas⁺) cell populations, suggesting a shift toward an anti-inflammatory immune profile (Figures 5 and 6). Figure 7 illustrates the treatment timeline of the patient.

Comprehensive immunological assessments were performed at three time points: prior to the initiation of molecular hydrogen therapy (November 13, 2024) for 2 months; 10 days of hydrogen with rhodiola therapy (from January 13 to 23, 2025); and 4 months post-hydrogen and hydrogen rhodiola therapy (March 13, 2025). The analyses focused on both T-cell and B-cell subsets, with particular emphasis on regulatory populations and the expression of immune checkpoint molecules, to evaluate the immunomodulatory effects of hydrogen therapy over time.

Following the initiation of molecular hydrogen therapy alone or in combination with rhodiola, the patient demonstrated gradual and sustained neurological improvement. By February 2025, left upper limb muscle strength had improved from MRC grades 1 to 3, and left lower limb strength similarly improved from grade 1 to 3. The NIHSS score improved from 12 to 10, indicating partial neurological recovery. These clinical improvements were paralleled by notable immunological changes (Figure 5). Specifically, there was a progressive increase in type 1 regulatory T cells (Tr1), rising from 40% at baseline to 90% at four months post-therapy. TIM-3 expression on cytotoxic T cells (Tc) increased by 40%, and a marked expansion of regulatory B-cell populations was observed. In addition, expression patterns of programmed cell death protein 1 (PD-1) on naïve helper (Th) and Tc T cells exhibited dynamic modulation over the course of treatment (Figures 5 and 6), suggesting an evolving immune-regulatory environment associated with recovery.

Immunophenotyping analyses were performed at three distinct time points in chronological order: on November 13, during the episode of arterial embolism; on January 13, 2 months after initiating hydrogen capsule treatment; and on January 23, 10 days after the addition of hydrogen-rhodiola capsules). During the right carotid artery embolism on November 13, an increase was observed in naïve and central memory (CM) Th cells, as well as in naïve Tc cells expressing KLRG1. Additionally, there was an elevation in Fas expression on marginal zone B cells and increased Fas expression in switched-memory (SM) and double-negative (DN) B cells. Levels of activated and natural regulatory T (Treg) cells, along with Tr1 cells, were also significantly higher (Figure 6).

Conversely, PD-1 expression decreased on naïve Th and naïve Tc cells, and TIM-3 expression was reduced on effector Th and Tc cells. Following the initiation of hydrogen capsule therapy on November 13 and subsequent STA–MCA bypass surgery on November 24, there was a reduction in naïve Th cells and CM cells expressing KLRG1, as well as a decline in activated natural Treg and Tr1 cells. By January 23, 10 days after the patient began hydrogen-rhodiola capsules, an increase in effector Th and Tc cells expressing TIM-3 was observed. Additionally, there was a decrease in naïve Tc cells expressing KLRG1, as well as a reduction in Fas-positive marginal zone B cells, SM B cells, and DN B cells (Figure 6).

By March 2025, after 4 months of molecular hydrogen-rhodiola therapy, the patient showed further neurological improvement. Left upper limb muscle strength increased to MRC grade 5, and left lower limb strength improved to MRC grade 4. Follow-up brain MRI on March 2, 2025, revealed no new ischemic or hemorrhagic lesions. Throughout the treatment period, no adverse effects associated with hydrogen therapy were reported. For further visualization, a dynamic stereoscopic video of the blood vessel model is available at the following link: https://sketchfab.com/3d-models/blood-vessel-09f09ebc66e14e2dabebbe9514a7a88a.

## 3 Discussion

This case report provides preliminary insights into the potential role of molecular hydrogen therapy alone or in combination with rhodiola as an adjuvant intervention for ischemic stroke secondary to ICA occlusion. Our findings suggest that hydrogen supplementation may support neurological recovery, in part, through modulation of the immune response, particularly by enhancing populations of regulatory immune cells that are known to promote tissue repair and resolve inflammation. Following the initiation of molecular hydrogen-rhodiola therapy, the patient exhibited marked clinical improvement, including substantial recovery of motor function that exceeded typical expectations for a severe stroke of this nature. Although natural recovery processes and concurrent medical interventions undoubtedly contributed to this outcome, the temporal correlation between hydrogen administration, immunological changes, and accelerated neurological recovery supports a possible therapeutic contribution from hydrogen-rhodiola supplementation.

The immunological findings are of particular interest. We observed a notable increase in Tr1 cells, a subset of T cells characterized by their production of anti-inflammatory cytokines such as interleukin-10 (IL-10) and transforming growth factor-beta (TGF-β). These cytokines are critical for creating an immunological milieu conducive to neural repair and suppression of secondary injury mechanisms [29]. In parallel, regulatory B cells (Bregs), which play an essential role in maintaining immune homeostasis and promoting resolution of inflammation [30], were also elevated during the course of therapy.

Another significant observation was the upregulation of TIM-3 expression on Tc cells. TIM-3 functions as an immune checkpoint molecule that suppresses overactive immune responses and contributes to peripheral tolerance [31]. Reduced TIM-3 expression has been implicated in the pathogenesis of various autoimmune diseases, including rheumatoid arthritis and multiple sclerosis, where immune regulation is impaired [32]. The observed increase in TIM-3 + Tc cells in this patient may reflect a shift toward a more controlled, anti-inflammatory immune state, thereby supporting recovery and limiting further neuronal injury. These immunological changes are consistent with evolving models of post-stroke immunology, which describe a biphasic immune response: an early pro-inflammatory phase followed by a regulatory or pro-resolving phase marked by expansion of immunosuppressive cell types [33]. Hydrogen therapy may facilitate or enhance this transition, thereby promoting neurorepair mechanisms and improving clinical outcomes. Moreover, rhodiola has also been previously reported to exhibit antioxidative, immunomodulatory, and neuroprotective properties, as well as modulatory effects on neurodegenerative processes [14,16,17,18].

While the exact pathways by which molecular hydrogen influences immune cell dynamics remain to be fully elucidated, several plausible mechanisms have been proposed. These include the scavenging of cytotoxic hydroxyl radicals and peroxynitrite, protection of mitochondrial integrity in immune cells, and inhibition of key pro-inflammatory signaling cascades such as nuclear factor kappa B [34,35]. Through these actions, hydrogen may indirectly support the proliferation and function of regulatory immune cells while mitigating the deleterious effects of oxidative stress. In addition, rhodiolin has been shown to inhibit inflammatory responses and oxidative stress through modulation of the phosphatidylinositol 3-kinase/protein kinase B signaling pathway [36,37].

Although the postoperative course was complicated by the development of a localized abscess requiring percutaneous drainage and a newly diagnosed tuberculosis infection, the patient demonstrated marked clinical improvement, with resolution of systemic symptoms and stabilization of vital signs, allowing for safe discharge with appropriate follow-up plans in place. Neurologically, there was a marked enhancement in consciousness level (Glasgow Coma Scale: E4V5M6) and full recovery of left upper limb motor strength (MRC grade 5/5). This case highlights the potential role of molecular hydrogen-rhodiola therapy as an adjuvant treatment for ischemic stroke, suggesting that its immunomodulatory effects may contribute to neurological recovery. The observed clinical improvements paralleled favorable immunological shifts, supporting its therapeutic potential in post-stroke management. However, *in vivo* studies have shown that salidroside, a key component of Tibetan medicine, enhances host defense against mycobacterial infection by boosting the production of inflammatory cytokines [19].

It is important to acknowledge that the patient presented with multiple comorbidities, including pulmonary tuberculosis, and underwent additional interventions such as drainage procedures, which may have influenced immune responses and neurological recovery. These factors represent significant potential confounders, making it difficult to attribute the observed clinical improvements solely to the adjuvant hydrogen and Rhodiola therapy. This case underscores the inherent complexity of interpreting therapeutic outcomes in a multifactorial clinical context.

Our findings are consistent with prior experimental studies and limited clinical observations that have demonstrated neuroprotective and anti-inflammatory effects of hydrogen therapy in models of cerebral ischemia [38,39]. However, this report contributes novel insights by providing serial, immunologically focused assessments that help to elucidate potential cellular mechanisms underlying clinical improvement.

Several limitations must be acknowledged in interpreting the findings of this case report. As a single-patient observation, it is not possible to establish causality between molecular hydrogen-rhodiola therapy and the clinical or immunological improvements observed. Furthermore, the extracranial–intracranial bypass surgery performed prior to the initiation of hydrogen therapy likely contributed to cerebral reperfusion and may have played a role in the observed clinical recovery. Although the patient exhibited notable clinical and immunological improvement following the administration of adjuvant molecular hydrogen and *Rhodiola rosea* therapy, it remains uncertain to what extent these improvements can be attributed to the treatment itself. Given that the patient also underwent a successful bypass surgery, the observed recovery may be the result of multiple contributing factors. Nonetheless, the temporal association between the therapy and the improvement, along with supportive changes in laboratory and immune markers, suggest a potential benefit. We recognize that conclusions regarding efficacy cannot be drawn from a single case, and further studies involving a larger number of patients are needed to validate these preliminary observations.

## 4 Conclusion

This case report underscores the potential utility of molecular hydrogen therapy alone or in combination with rhodiola as an adjuvant treatment for ischemic stroke, particularly in the context of ICA occlusion. The observed expansion of regulatory immune cell populations, including Tr1 and Breg cells, along with increased TIM-3 expression on cytotoxic T cells, suggests a possible immunomodulatory mechanism contributing to neurological recovery. These preliminary findings support the need for further investigation through well-designed clinical trials to validate the therapeutic efficacy, determine optimal dosing regimens, and elucidate the underlying molecular mechanisms of hydrogen therapy alone or in combination with rhodiola in the management of cerebrovascular diseases.

## References

[1] Goyal M, Menon BK, van Zwam WH, Dippel DW, Mitchell PJ, Demchuk AM, et al. Endovascular thrombectomy after large-vessel ischaemic stroke: a meta-analysis of individual patient data from five randomised trials. Lancet. 2016;387:1723–31. 10.1016/S0140-6736(16)00163-X 26898852

[2] Powers WJ, Rabinstein AA, Ackerson T, Adeoye OM, Bambakidis NC, Becker K, et al. Guidelines for the early management of patients with acute ischemic stroke: 2019 update to the 2018 guidelines for the early management of acute ischemic stroke: a guideline for healthcare professionals from the american heart association/american stroke association. Stroke. 2019;50:e344–418. 10.1161/STR.0000000000000211 31662037

[3] Nogueira RG, Jadhav AP, Haussen DC, Bonafe A, Budzik RF, Bhuva P, et al. Thrombectomy 6 to 24 hours after stroke with a mismatch between deficit and infarct. N Engl J Med. 2018;378:11–21. 10.1056/NEJMoa1706442 29129157

[4] Chamorro Á, Dirnagl U, Urra X, Planas AM. Neuroprotection in acute stroke: targeting excitotoxicity, oxidative and nitrosative stress, and inflammation. Lancet Neurol. 2016;15:869–1. 10.1016/S1474-4422(16)00114-9 27180033

[5] Amin-Hanjani S, Barker 2nd FG, Charbel FT, Connolly Jr ES, Morcos JJ, Thompson BG. Extracranial-intracranial bypass for stroke-is this the end of the line or a bump in the road? Neurosurgery. 2012;71:557–61. 10.1227/NEU.0b013e3182621488 22668888

[6] Kwakkel G, Kollen BJ, Krebs HI. Effects of robot-assisted therapy on upper limb recovery after stroke: a systematic review. Neurorehabil Neural Repair. 2008;22:111–21. 10.1177/1545968307305457 PMC2730506 17876068

[7] Ohsawa I, Ishikawa M, Takahashi K, Watanabe M, Nishimaki K, Yamagata K, et al. Hydrogen acts as a therapeutic antioxidant by selectively reducing cytotoxic oxygen radicals. Nat Med. 2007;13:688–94. 10.1038/nm1577 17486089

[8] Ohta S. Molecular hydrogen as a novel antioxidant: overview of the advantages of hydrogen for medical applications. Methods Enzymol. 2015;555:289–317. 10.1016/bs.mie.2014.11.038 25747486

[9] Ishibashi T. Molecular hydrogen: new antioxidant and anti-inflammatory therapy for rheumatoid arthritis and related diseases. Curr Pharm Des. 2013;19:6375–81. 10.2174/13816128113199990507 PMC3788323 23859555

[10] Saengsin K, Sittiwangkul R, Chattipakorn SC, Chattipakorn N. Hydrogen therapy as a potential therapeutic intervention in heart disease: from the past evidence to future application. Cell Mol Life Sci. 2023;80:174. 10.1007/s00018-023-04818-4 PMC10239052 37269385

[11] Chen JY, Lu JW, Feng SW, Ho YJ, Lui SW, Hsieh TY, et al. Molecular hydrogen therapy in aneurysmal SAH With RA and newly-diagnosed SLE, complicated with acute ischemic infarction: a case report of improved immune markers including Tr1 cells, breg cells and TIM3 expression on Tc cells. In Vivo. 2024;38:3131–7. 10.21873/invivo.13799 PMC11535933 39477420

[12] Kumagai K, Toyooka T, Takeuchi S, Otani N, Wada K, Tomiyama A, et al. Hydrogen gas inhalation improves delayed brain injury by alleviating early brain injury after experimental subarachnoid hemorrhage. Sci Rep. 2020;10:12319. 10.1038/s41598-020-69028-5 PMC7378202 32704088

[13] Chen H, Xie K, Han H, Li Y, Liu L, Yang T, et al. Molecular hydrogen protects mice against polymicrobial sepsis by ameliorating endothelial dysfunction via an Nrf2/HO-1 signaling pathway. Int Immunopharmacol. 2015;28:643–54. 10.1016/j.intimp.2015.07.034 26253656

[14] Pu WL, Zhang MY, Bai RY, Sun LK, Li WH, Yu YL, et al. Anti-inflammatory effects of Rhodiola rosea L.: A review. Biomed Pharmacother. 2020;121:109552. 10.1016/j.biopha.2019.109552 31715370

[15] Zhang L, Yin H, Xie Y, Zhang Y, Dong F, Wu K, et al. Exploring the anti‑oxidative mechanisms of Rhodiola rosea in ameliorating myocardial fibrosis through network pharmacology and in vitro experiments. Mol Med Rep. 2024;30. 10.3892/mmr.2024.13338 PMC11450433 39370810

[16] Recio MC, Giner RM, Manez S. Immunmodulatory and antiproliferative properties of rhodiola species. Planta Med. 2016;82(11–12):952–60. 10.1055/s-0042-107254 27224273

[17] Lee Y, Jung JC, Jang S, Kim J, Ali Z, Khan IA, et al. Anti-Inflammatory and Neuroprotective Effects of Constituents Isolated from Rhodiola rosea. Evid Based Complement Altern Med. 2013;2013:514049. 10.1155/2013/514049 PMC3652169 23690847

[18] Zhang X, Wang X, Hu X, Chu X, Li X, Han F. Neuroprotective effects of a Rhodiola crenulata extract on amyloid-beta peptides (Abeta(1-42)) -induced cognitive deficits in rat models of Alzheimer’s disease. Phytomedicine. 2019;57:331–8. 10.1016/j.phymed.2018.12.042 30807987

[19] He S, Fan H, Sun B, Yang M, Liu H, Yang J, et al. Tibetan medicine salidroside improves host anti-mycobacterial response by boosting inflammatory cytokine production in zebrafish. Front Pharmacol. 2022;13:936295. 10.3389/fphar.2022.936295 PMC9470765 36120339

[20] Iadecola C, Anrather J. The immunology of stroke: from mechanisms to translation. Nat Med. 2011;17:796–808. 10.1038/nm.2399 PMC3137275 21738161

[21] Tian Y, Zhang Y, Wang Y, Chen Y, Fan W, Zhou J, et al. Hydrogen, a novel therapeutic molecule, regulates oxidative stress, inflammation, and apoptosis. Front Physiol. 2021;12:789507. 10.3389/fphys.2021.789507 PMC8721893 34987419

[22] Lui SW, Lu JW, Ho YJ, Tang SE, Ko KH, Hsieh TY, et al. Molecular hydrogen as a promising therapy could be linked with increased resting treg cells or decreased Fas + T cell subsets in a IgG4-PF-ILD patient: a case report. In Vivo. 2024;38:1512–8. 10.21873/invivo.13600 PMC11059909 38688598

[23] Lin YT, Lu JW, Lin JC, Ho YJ, Lui SW, Hsieh TY, et al. Molecular hydrogen capsule therapy for primary biliary cholangitis with elevated IgG4: a case report on immune marker normalization. In Vivo. 2025;39:1669–75. 10.21873/invivo.13968 PMC12042006 40295001

[24] Tu TH, Lu JW, Wu CH, Ho YJ, Lui SW, Hsieh TY, et al. Molecular hydrogen therapy for SLE-PAH: case report on immune marker modulation. In Vivo. 2025;39:1211–9. 10.21873/invivo.13926 PMC11884449 40010970

[25] Lin YT, Lu JW, Ho YJ, Lui SW, Hsieh TY, Liu HC, et al. Molecular hydrogen as an adjuvant therapy in severe lupus serositis with heart failure: a case report on immune modulation and fatigue reduction. In Vivo. 2025;39:1200–6. 10.21873/invivo.13924 PMC11884437 40010949

[26] Hsu HF, Hu RY, Lu JW, Hueng DY, Ho YJ, Lui SW, et al. Molecular hydrogen therapy enhances immune markers in treg, plasma, Tr1 Cells, and KLRG1 expression on Tc cells: a case of acute SDH with midline shift and uncal herniation post-decompressive craniectomy. In Vivo. 2025;39:1190–9. 10.21873/invivo.13923 PMC11884442 40010997

[27] Lin YT, Lu JW, Ho YJ, Lui SW, Hsieh TY, Wang KY, et al. Molecular hydrogen as a potential adjunctive therapy to improve renal function and reduce fatigue in an elderly patient with chronic comorbidities: a case report. In Vivo. 2025;39:572–6. 10.21873/invivo.13862 PMC11705128 39740897

[28] Lui SW, Hsieh TY, Lu JW, Chen YC, Lin TC, Ho YJ, et al. Predicting the clinical efficacy of JAK inhibitor treatment for patients with rheumatoid arthritis based on Fas + T cell subsets. APMIS. 2023;131:498–509. 10.1111/apm.13341 37439387

[29] Freeborn RA, Strubbe S, Roncarolo MG. Type 1 regulatory T cell-mediated tolerance in health and disease. Front Immunol. 2022;13:1032575. 10.3389/fimmu.2022.1032575 PMC9650496 36389662

[30] Zhu Q, Rui K, Wang S, Tian J. Advances of regulatory B cells in autoimmune diseases. Front Immunol. 2021;12:592914. 10.3389/fimmu.2021.592914 PMC8082147 33936028

[31] Wolf Y, Anderson AC, Kuchroo VK. TIM3 comes of age as an inhibitory receptor. Nat Rev Immunol. 2020;20:173–85. 10.1038/s41577-019-0224-6 PMC7327798 31676858

[32] Das M, Zhu C, Kuchroo VK. Tim-3 and its role in regulating anti-tumor immunity. Immunol Rev. 2017;276:97–111. 10.1111/imr.12520 PMC5512889 28258697

[33] Anrather J, Iadecola C. Inflammation and stroke: an overview. Neurotherapeutics. 2016;13:661–70. 10.1007/s13311-016-0483-x PMC5081118 27730544

[34] Iuchi K, Nishimaki K, Kamimura N, Ohta S. Molecular hydrogen suppresses free-radical-induced cell death by mitigating fatty acid peroxidation and mitochondrial dysfunction. Can J Physiol Pharmacol. 2019;97:999–1005. 10.1139/cjpp-2018-0741 31295412

[35] Ishihara G, Kawamoto K, Komori N, Ishibashi T. Molecular hydrogen suppresses superoxide generation in the mitochondrial complex I and reduced mitochondrial membrane potential. Biochem Biophys Res Commun. 2020;522:965–70. 10.1016/j.bbrc.2019.11.135 31810604

[36] Bo J, Mao S, Yang J, Wang L, Zheng J, Zhang C, et al. Rhodiolin inhibits the PI3K/AKT/mTOR signaling pathway via the glycolytic enzyme GPI in human papillary thyroid cancer. Phytomedicine. 2024;132:155804. 10.1016/j.phymed.2024.155804 38943696

[37] Jiang L, Yang D, Zhang Z, Xu L, Jiang Q, Tong Y, et al. Elucidating the role of Rhodiola rosea L. in sepsis-induced acute lung injury via network pharmacology: emphasis on inflammatory response, oxidative stress, and the PI3K-AKT pathway. Pharm Biol. 2024;62:272–84. 10.1080/13880209.2024.2319117 PMC10919309 38445620

[38] Eckermann JM, Chen W, Jadhav V, Hsu FP, Colohan AR, Tang J, et al. Hydrogen is neuroprotective against surgically induced brain injury. Med Gas Res. 2011;1:7. 10.1186/2045-9912-1-7 PMC3231979 22146427

[39] Ono H, Nishijima Y, Adachi N, Sakamoto M, Kudo Y, Kaneko K, et al. A basic study on molecular hydrogen (H2) inhalation in acute cerebral ischemia patients for safety check with physiological parameters and measurement of blood H2 level. Med Gas Res. 2012;2:21. 10.1186/2045-9912-2-21 PMC3457852 22916706

